# Environmental Nutrients Alter Bacterial and Fungal Gut Microbiomes in the Common Meadow Katydid, *Orchelimum vulgare*

**DOI:** 10.3389/fmicb.2020.557980

**Published:** 2020-10-23

**Authors:** Melani Muratore, Yvonne Sun, Chelse Prather

**Affiliations:** Department of Biology, University of Dayton, Dayton, OH, United States

**Keywords:** insect microbiome, fungal microbiome, nutrient limitation, bacterial microbiome, katydid, grasshopper, *Orchelimum vulgare*

## Abstract

Insect gut microbiomes consist of bacteria, fungi, and viruses that can act as mutualists to influence the health and fitness of their hosts. While much has been done to increase understanding of the effects of environmental factors that drive insect ecology, there is less understanding of the effects of environmental factors on these gut microbial communities. For example, the effect of environmental nutrients on most insect gut microbiomes is poorly defined. To address this knowledge gap, we investigated the relationship between environmental nutrients and the gut microbial communities in a small study of katydids (*n* = 13) of the orthopteran species *Orchelimum vulgare* collected from a costal prairie system. We sampled *O. vulgare* from unfertilized plots, as well as from plots fertilized with added nitrogen and phosphorus or sodium separately and in combination. We found significantly higher Shannon diversity for the gut bacterial communities in *O. vulgare* from plots fertilized with added sodium as compared to those collected from plots without added sodium. In contrast, diversity was significantly lower in the gut fungal communities of grasshoppers collected from plots with added nitrogen and phosphorus, as well as those with added sodium, in comparison to those with no added nutrients. There was also a strong positive correlation between the gut bacterial and gut fungal community diversity within each sample. Indicator group analysis for added sodium plots included several taxa with known salt-tolerant bacterial and fungal representatives. Therefore, despite the small sample number, these results highlight the potential for the gut bacterial and fungal constituents to respond differently to changes in environmental nutrient levels. Future studies with a larger sample size will help identify mechanistic determinants driving these changes. Based on our findings and the potential contribution of gut microbes to insect fitness and function, consideration of abiotic factors like soil nutrients along with characteristic gut microbial groups is necessary for better understanding and conservation of this important insect herbivore.

## Introduction

Recently, the rise in the research interest on insect microbiomes has helped reveal novel insights and understanding of insect ecology. Both resident and transient microbes exhibited effects on the health and fitness of their insect hosts (McKenney et al., 2018), such as improved nutrition (Brune, 2014; Paniagua Voirol et al., 2018), changes to host behaviors (Dillon et al., 2000), increased reproductive success (Kaltenpoth et al., 2009), and protection from environmental pathogens and pesticides (Paniagua Voirol et al., 2018). Conversely, behaviors of the insect host like feeding and social roles can be reflected in variations in their microbiome (Yun et al., 2014; Jones et al., 2018; Kakumanu et al., 2018; Obadia et al., 2018). Recently, we demonstrated that orthopterans, specifically katydids and grasshoppers, share a characteristic bacterial community dominated by Proteobacteria, Firmicutes, and Actinobacteria (Muratore et al., 2020). Because these organisms were collected from a coastal prairie under a fully factorial fertilization experiment, we had a unique opportunity to further dissect how the microbial community composition responds to nutrient modifications in the environment.

When an organism is limited by a particular nutrient, it is expected that an addition of that nutrient to the environment will result in increased biomass of that organism (van der Ploeg et al., 1999). For example, when plants are experiencing nitrogen and phosphorus limitation, the addition of nitrogen and phosphorus to the soil results in increased plant biomass. At the community level, in contrast, when an essential nutrient like nitrogen is increased, fast-growing, nitrophilic plant species often increase in abundance to the exclusion of other slower growing species (Southon et al., 2013), resulting in declines in plant community richness and diversity (Clark and Tilman, 2008; Soons et al., 2017; Midolo et al., 2019). Despite our understanding in how plant communities respond to nutrient limitation and subsequent increases, how consumer communities respond to nutrient fluctuations is less established. An early field study found that fertilization of grasses with ammonium nitrate in a Nebraska prairie resulted in increases in both the nitrogen abundance in the foliage as well as the biomass of grasshoppers (Heidorn and Joern, 1987). Similarly in a feeding trial study, grasshoppers fed with nitrogen- or phosphorus-enriched grasses from a Kansas tallgrass prairie ecosystem showed increased growth rates (Rode et al., 2017). At our field study in a Texas coastal prairie, grasshoppers also showed an increase in abundance in plots treated with nitrogen, phosphorus, and sodium, an observation indicative of nutrient co-limitation experienced by the herbivores ecosystem (Prather et al., 2018). However, a concomitant increase in the herbivore richness and diversity was also observed in this ecosystem (Prather et al., 2018). While specific mechanisms, such as nutrient status of plants and soil or insect feeding behavior, underlying these observations remain to be determined, it is clear that environmental perturbations of nutrients may indeed affect grasshopper ecology and potentially reflect the nutrient status experienced by these animals in a manner that does not fully follow the conventional wisdom with plants.

In addition to nutrients like nitrogen and phosphorus, sodium plays a key role in insect ecology. While typically considered non-essential for most plants (Maathuis, 2014), sodium is essential for animal physiology, controlling the osmolarity of body fluids (Geerling and Loewy, 2008) and regulating growth and reproduction. Sodium is also crucial for proper nervous system function (Liebeskind et al., 2011). Animals, including insects, have been known to satisfy their needs for sodium through activities beyond normal feeding behaviors (Smedley and Eisner, 1995; Morris et al., 2008). Grassland plant consumers, with limited sodium present in their food source, potentially experience sodium limitation and may exhibit sodium-seeking behaviors (Kaspari et al., 2017; Welti et al., 2019). Sodium levels in the soil can change naturally due to sodium carried by ocean winds in a coastal system, or anthropogenically by the additions of chemicals like ice-melting road salts (Franzén, 1990; Snell-Rood et al., 2014). Our study showing that grasshopper abundance and diversity were significantly higher in areas where sodium, nitrogen, and phosphorus were added strongly argues for nutrient colimitation in these animals and highlights the importance of sodium in a high-sodium grassland ecosystem (Prather et al., 2018).

*Orchelimum vulgare* is an insect herbivore that plays pivotal roles in the grassland ecosystems of North America (Báldi and Kisbenedek, 1997; Branson et al., 2006). *O. vulgare* is a key consumer of plant biomass as well as a key food source for predators – thus contributing to nutrient cycling and plant community composition in grasslands (Prather et al., 2017). Therefore, it has both economic and ecological significance. Though primarily feeding on plants, *O. vulgare* has been observed feeding on carcasses of insects and other small animals (Campbell et al., 2010; Zeng et al., 2016). The flexible feeding behavior might affect the composition of the gut microbiome, which can further impact the animal’s fitness and functions. While our earlier work identified a “core” bacterial community in the gut of *O. vulgare* (Muratore et al., 2020), whether the microbiome composition of *O. vulgare* can be modulated by environmental nutrient conditions has not been determined.

In this study, we used a small cohort of samples (3–4 individuals per nutrient condition) to begin to explore the impact of environmental nutrient conditions on the gut microbiome of *O. vulgare* by analyzing the bacterial and fungal gut microbiome of individuals collected from large experimental treatment plots in a coastal tallgrass prairie. These treatment plots were amended with two different fertilizer treatments – nitrogen and phosphorus together (NP) and sodium alone (Na) each at two levels (ambient or added). If the gut microbiomes in *O. vulgare* experienced nutrient limitation and subsequently responded to added nutrient treatments, their compositions would be expected to change in individuals collected from added nutrient plots than those collected from ambient plots. More specifically, if the gut microbial communities responded to added nutrients similarly as their hosts, we would expect to see a significant increase in microbial diversity. Alternatively, if the gut microbial communities responded to added nutrients similarly as plant communities, we would expect to see a significant decrease in microbial diversity.

## Materials and Methods

### Sample Collection

*Orchelimum vulgare* were collected from a coastal prairie, which is part of a large-scale fertilization experiment to study orthopteran communities at the University of Houston’s Coastal Center near Houston, Texas. Many orthopteran species have been documented in this prairie, of which *O. vulgare* is one of the most common members (Prather et al., 2018). This species is an omnivore, and eats a mixed diet of plants and insect prey. The prairie topography is generally flat with a maximum of 2 cm gradient in elevation separating the experimental plots. The experimental site follows a fully factorial design manipulating nitrogen and phosphorus (N and P together at two levels, ambient and added) and sodium (Na at two different levels, ambient or added) with eight replicates in each treatment (*n* = 2 levels of NP × 2 different levels of Na × 8 replicates = 32 experimental plots). The prairie was divided into large plots (30 × 30 m^2^) which were subsequently treated with fertilizers. Fertilizers were applied in March of 2016 and 2017 before the beginning of the growing season. We added nitrogen (in the form of urea) and phosphorus (in the form of monoammonium phosphate) and sodium at rates of 10 g/m^2^ to bring the top 10 cm of soil to approximately 30% higher than ambient levels (Heidorn and Joern, 1987; Southon et al., 2013). We collected *O. vulgare* individuals from as many replicates as possible via sweep-netting during 1-day of sampling in June of 2017. Our sample size was limited by the number of individuals we caught in each plot that day, which was determined by local abundance at the time. In total we included 13 *O. vulgare* individuals at 4th instar or later in development in this study: three individuals from plots with no added nutrients (None), four individuals from plots treated only with sodium (Na), three from plots treated with only nitrogen and phosphorus (NP), and three from plots treated with nitrogen and phosphorus and sodium (NP × Na). The insect samples were shipped on ice to the University of Dayton (Dayton, OH) and stored frozen at −20°C until dissection. Dissection consisted of removal of the entire gut, including contents, from the crop to the hindgut using instruments sterilized in 95% (v/v) lab-grade ethanol between each dissection. Gut samples were stored at −20°C until DNA extraction.

### DNA Extraction

A detailed description of this procedure was explained elsewhere (Muratore et al., 2020). Briefly, frozen *O. vulgare* gut samples were homogenized into smaller pieces for DNA extraction using the Qiagen DNeasy Blood and Tissue Kit (Qiagen 69504) following the manufacturer’s protocol. Concentration of total DNA in each sample was measured by a nanophotometer (Implen, Denville Scientific Inc.) and then the extracted DNA sample was stored at −20°C until sequencing.

### Small Subunit rRNA Gene Sequencing and Identification

As explained in a previous publication (Muratore et al., 2020), high throughput DNA sequencing was performed by Zymo Research (Irvine, CA). Before the library construction took place, extracted DNA was quantified using nanodrop and the 2100 Bioanalyzer System (Agilent). The V3–V4^TM^ region of the bacterial and Archaeal 16S rRNA gene which was amplified using the Quick-16S Primer Set V3-V4 (Zymo Research, Irvine, CA). The ITS2 region in fungal species was amplified using the ZymoBIOMICS Services ITS2 Primer Set.

The sequencing library prepared by Zymo utilized real-time PCR to prevent chimera formation and to control cycles. The PCR products were quantified with qPCR fluorescence readings. These products were pooled together based on equal molarity, and the library was cleaned up with Select-a-Size DNA Clean & Concentrator^TM^, then subsequently quantified with TapeStation^®^ and Qubit^®^. The libraries were sequenced on the Illumina HiSeq2500 platform in “Rapid Run” mode with a v3 reagent kit (600 cycles), using 100 bp paired end sequencing, with an average of 10.2 million reads per sample. Samples were collected in 4 cycle intervals until sufficient amplification had occurred, or processing was ended at 42 cycles if no amplification occurred. PCR Single nucleotide differences were distinguished among sequences and used along with the Greengenes database (gg_13_8) to establish taxonomic identification. Unique amplicon sequences were inferred, and chimeras removed using the DADA2 pipeline (Callahan et al., 2016). Taxonomy assignments were made using Uclust from Qiime (v.1.9.1) (Caporaso et al., 2010) and an internally curated research database (Zymo). Information about the number of reads per sample is listed in the Supplementary Table S1. The rarefaction curves of bacterial and fungal species for each sample as well as the species accumulation curves are shown in the Supplementary Figures S1, S2.

### Data Analysis

Statistical analysis was performed in R (version 3.6.2). Diversity calculations, including Shannon and Inverse Simpson diversity as well as richness, were performed using the *vegan* package in R. To test for differences between treatments, we used ANOVA with two independent variables (NP or Na) at two levels (ambient and added) at the species level. To look at effect sizes, we calculated Cohen’s d using the cohen.d function in the *effsize* package in R at the species level. Regression comparing bacterial and fungal diversity was carried out in base R. Non-metric multidimensional scaling (NMDS) was performed at the species level using the *vegan* package in R. NMDS plots were used if the model arrived at convergence. Analysis of similarity (ANOSIM) was used to measure Bray-Curtis dissimilarity, corresponding with the NMDS plots was also performed using R. Indicator values were calculated using the indval function in the *labdsv* package in R. All indicator values specified had a *p* value of less than 0.05. Species accumulation was calculated using the specaccum function in the *vegan* package of R. In order to highlight dominant groups, bacterial groups are classified as “other” when they are present at less than 2% of average relative abundance in at least two treatment groups and did not appear in every treatment with the exception of Entomoplasmatales families, which appeared in only three treatments but at high relative abundance. Fungal orders are classified as “other” when they are present at less than 2% of average relative abundance in at least two treatments and did not appear in all treatments.

## Results

### Bacterial Communities

**FIGURE 1:**
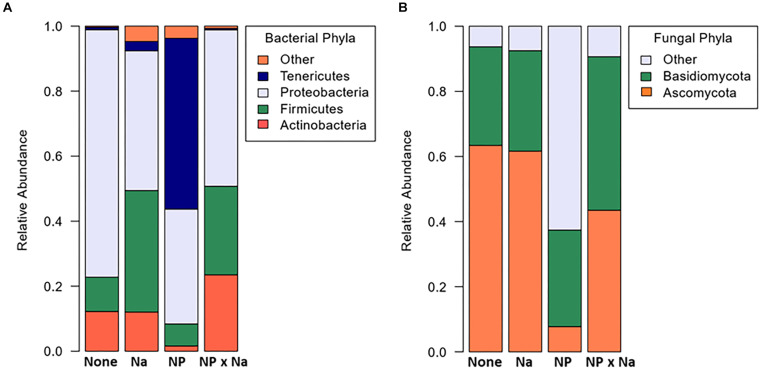
Average relative abundance of microbial phyla in grasshopper samples from four treatments. The four treatments include: no added nutrients or “None” (*n* = 3), added sodium or “Na” (*n* = 4), added nitrogen and phosphorus or “NP” (*n* = 3), and nitrogen and phosphorus added with sodium or “NP × Na” (*n* = 3). The “Other” category is comprised of groups that do not appear in all four treatments and comprise less than 2% of reads. **(A)** Relative abundance of bacterial phyla. **(B)** Relative abundance of fungal phyla.

**FIGURE 2:**
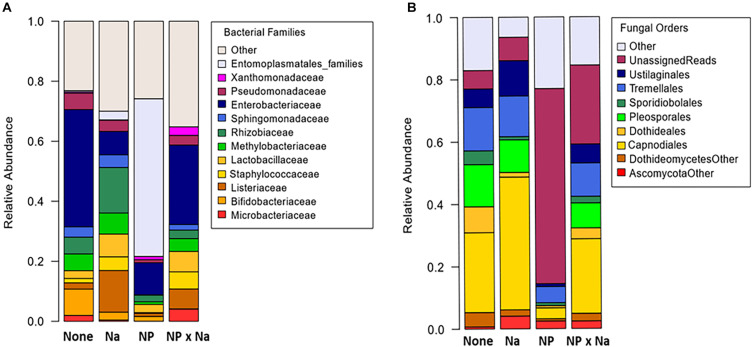
Average relative abundance of microbial taxa in grasshopper samples from four treatments. The four treatments include: no added nutrients or “None” (*n* = 3), added sodium or “Na” (*n* = 4), added nitrogen and phosphorus or “NP” (*n* = 3), and nitrogen and phosphorus added with sodium or “NP × Na” (*n* = 3). In all The “Other” category is comprised of groups that do not appear in all four treatments and comprise less than 2% of OTUs. The only exception is the Entomoplasmatales families included which were not present in the nitrogen and phosphorus with sodium treatment, but which were present in the other three treatments. **(A)** Relative abundance of bacteria families. **(B)** Relative abundance of fungal orders.

A total of 171 bacterial and 99 fungal species-level operational taxonomic units (OTUs) were identified in the 13 samples (Table 1). Curiously, no archaeal species were detected in any of the samples. A mean bacterial richness of 31.6 (± 3.9) was observed across 13 samples. Bacterial phyla present in all four treatments (None, NP, NP × Na, and Na) included Proteobacteria, Firmicutes, and Actinobacteria. While Proteobacteria was the most abundant phylum in all four treatment groups (Figure 1A), the most notable shift in bacterial phyla was observed in the predominance of Tenericutes in the NP treatment group. There was a total of 11 bacterial families present at an average relative abundance of 2% or higher in all four treatment groups (Figure 2A) with the 6 most abundant families being Enterobacteriaceae, Lactobacillaceae, Listeriaceae, Methylobacteriaceae, Pseudomonadaceae, and Rhizobacteriaceae. Uncategorized family OTUs from the order Entomoplasmatales were present in four out of the 13 samples, representing three of the four treatment groups. They comprise a large proportion of families in the NP treatment group but not the Na or NP × Na treatment groups. Five families, including Streptococcaceae, Propionibacteriaceae, Phyllobacteriaceae, Listeriaceae, and Corynebacteriaceae (indicator values, respectively: 0.9979, 0.9801, 0.9627, 0.8745, 0.7094), are clear indicators of bacterial communities in the Na treatment group. Only one of these, Listeriaceae, is found at high relative abundance. In contrast, only one family, Sphingomonadaceae, was an indicator of the NP treatment group (indicator value, 0.7986).

**TABLE 1:** Gut microbiome richness and diversity for four treatments.

Treatment group	Sample	Bacterial richness	Bacterial inverse simpson index	Bacterial shannon index	Fungal richness	Fungal inverse simpson index	Fungal shannon index
None	#1	40	10.35	2.81	42	10.58	2.94
	#2	40	3.18	1.74	50	6.5	2.61
	#3	17	6.15	2.23	22	5.63	2.04
NP	#4	21	4.19	2.12	24	2.12	1.39
	#5	19	2.13	1.18	21	1.36	0.75
	#6	7	1.17	0.39	7	1.01	0.04
NP × Na	#7	30	8.32	2.68	25	3.78	1.88
	#8	37	14.59	3.03	16	9.45	2.44
	#9	65	3.58	2.44	42	4.97	2.32
Na	#10	26	11.17	2.76	11	6.71	2.12
	#11	35	4.84	2.26	19	8.23	2.41
	#12	30	6.3	2.4	26	5.14	2.04
	#13	41	12.81	2.92	25	5.28	2.23

**FIGURE 3:**
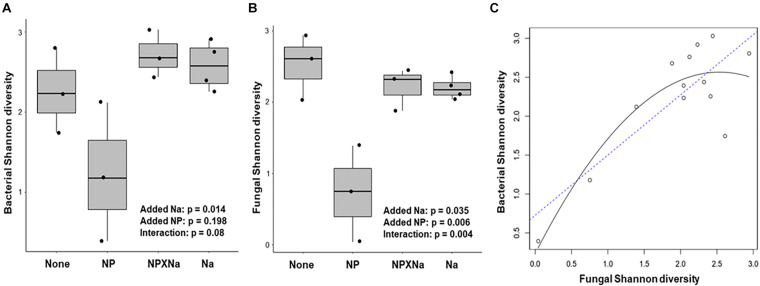
Analysis of diversity over four treatments. **(A)** Bacterial species Shannon diversity for each treatment was calculated with the *vegan* package in R. Two-way ANOVA in base R was used to compare levels of diversity between treatments and test for significant difference. Treatment effect size, or Cohen’s d, as calculated in R with the *effsize* package for Na compared to control was large (1.04). Effect size for NP was smaller (–0.32). **(B)** Fungal species Shannon diversity for each treatment was also calculated in a similar manner. Cohen’s d effect size for Na as compared to control was large (–1.12), as was NP compared to control (–1.28). **(C)** Regression analysis performed in R indicates a significant positive correlation between bacterial Shannon diversity and Fungal Shannon diversity among all 13 samples (*R*^2^ = 0.75).

Alpha diversity of bacterial communities was assessed in terms of taxa richness, Shannon Index, and Inverse Simpson Index (Table 1). Shannon diversity of bacterial taxa assessed at the species level (Figure 3A) was significantly higher for gut samples from the NP treatment group (*p* = 0.014) with a large treatment effect size (Cohen’s *d* = 1.04) compared to individual communities from plots with no added nutrients. Moreover, there was a moderate, but not significant, interaction between added Na and added NP groups (*p* = 0.08).

**FIGURE 4:**
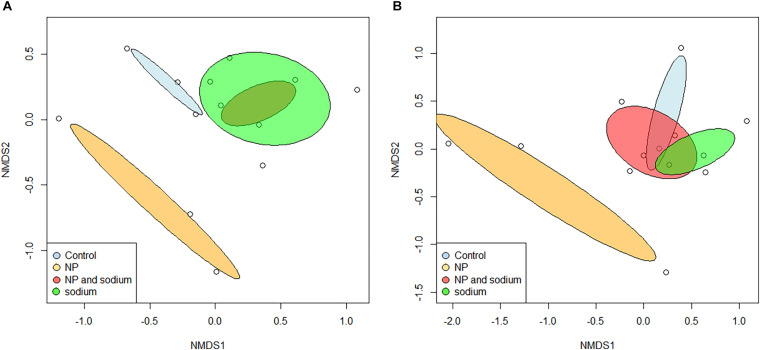
Nonmetric multidimensional scaling of grasshopper gut community assemblages at the family level (*n* = 13) was plotted using the vegan package in R. Dispersion ellipses are placed for each treatment group. Bray-Curtis dissimilarity was well-preserved in two dimensions. **(A)** Bacterial characterization. Analysis of Similarity (ANOSIM) was performed with a Bray-Curtis dissimilarity measure and showed an overall significant difference in bacterial families among treatments (*R* = 0.299). **(B)** Fungal characterization. ANOSIM was performed with a Bray-Curtis dissimilarity measure and showed an overall significant difference in fungal families among treatments (*R* = 0.335).

**FIGURE 5:**
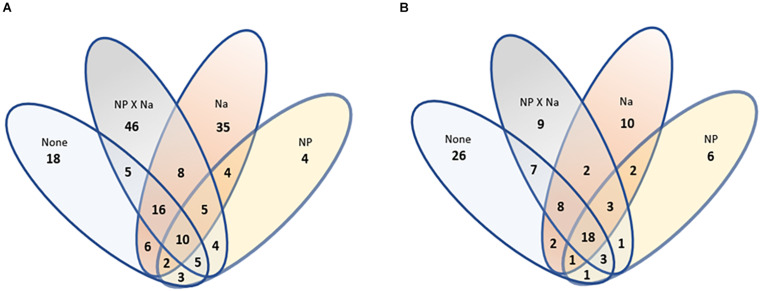
An analysis of shared groups among the four treatments. **(A)** A Venn diagram of bacterial species richness. Total number of bacterial species OTUs = 171. **(B)** A Venn diagram of fungal species richness. Total number of fungal species OTUs = 99.

Beta diversity of bacterial communities was assessed using non-metric multidimensional scaling (NMDS) performed at the species level (Figure 4A). There appeared to be an overlap in characteristic community between the control group (None) and the two added Na treatment groups (Na and NP × Na). The characteristic communities of the NP treatment group shared less similarity with the characteristic communities of the other three treatments (ANOSIM, *R* = 0.299). Beta diversity was further characterized by assessment of shared species level OTUs between treatment groups (Figure 5A), revealing that 60% of total bacterial species level OTUs were unique to each treatment group. Conversely, only 5.8% of bacterial OTUs were shared amongst all four treatment groups. The relative abundance of unique bacterial OTUs were notably higher in added Na treatment groups, at 46.5% (NP × Na) and 40.7% (Na), compared to 27.6% (None) and 10.8% (NP) in no added Na groups.

### Fungal Communities

A total of 99 OTUs were identified as fungal organisms with a mean fungal richness of 25.4 (± 3.3) among the 13 samples. The two most dominant fungal phyla in all four treatments were Ascomycota and Basidiomycota, but a relatively high number of fungal OTUs in the NP treatment group were unassigned (Figure 1B). Fungal data had a high number of specified OTUs at the order level of taxonomic classification (Figure 2B). Again, there was a high number of unspecified fungal OTUs for the NP treatment group even at order level. There were six predominant fungal orders that appeared in every treatment group: Capnodiales, Dothideales, Pleosporales, Sporidiobolales, Tremellales, and Ustilaginales. An unspecified species of *Cladosporium* was indicative of the Na treatment group (indicator value = 0.7806).

Alpha diversity of fungal communities was also assessed in terms of taxa richness, Shannon Index, and Inverse Simpson Index (Table 1). Shannon diversity of fungal species (Figure 3B) was significantly lower in the added Na treatment groups (Na and NP × Na, *p* = 0.035) and the added NP treatment groups (NP and NP × Na, *p* = 0.006), with a large negative treatment effect (Cohen’s *d* = −1.12 and −1.28, respectively). Notably for fungal communities, there was a significant interaction between added Na and added NP (*p* = 0.004). More specifically, the interaction between added NP and added Na in the NP × Na treatment group restored fungal diversity present in the NP treatment group to the same level as the Na treatment group. Regression analysis (Figure 3C) of bacterial species diversity versus fungal species diversity showed a strong positive correlation between the two indices (*R*^2^ = 0.75, *p* = 0.0006).

Beta diversity was also assessed using NMDS performed at the species level for fungi (Figure 4B). As with the bacterial communities, the characteristic communities of the NP treatment group again shared less similarity with the characteristic communities of the other three treatments (ANOSIM, *R* = 0.335). Beta diversity was also further characterized for fungal communities in terms of shared species with 51.5% of fungal species level OTUs unique to each treatment group (Figure 5B). In contrast, 18% of all fungal OTUs were shared amongst all four treatment groups. The control group (None) contains the highest number of unique OTUs (39.4%), compared to the NP × Na, NP, and Na treatment groups (39.4, 17.1, and 23.3, respectively).

## Discussion

Environmental nutrients significantly altered the gut bacterial and fungal communities in this exploratory study using a small number of field-collected katydids. These patterns of changes in bacterial and fungal diversity did not mimic the changes that we saw in grasshopper densities in this experiment (Prather et al., 2018). In particular, katydid densities increased when both NP and Na were added to soils. Here, bacterial community diversity declined with added NP, but increased with added Na, while fungal community diversity declined in response to both NP and Na. Despite the limited sample number, these contrasting observations in katydid densities and katydid gut microbiome argued for further investigations to better establish the connections between environmental nutrient perturbations and the ecology of the host animals with their gut microbiomes. Here, we would like to offer some interpretations of our preliminary findings by first considering the role NP and Na on plant communities as a functional intermediate.

NP affected the plant community in ways that could have altered grasshopper feeding and density. Plant community biomass was higher with added NP (Prather et al., 2018), and NP addition changed functional composition of the plant community (Prather et al, unpublished data). In contrast, while increases in Na led to higher concentrations of soil Na, this nutrient did not affect plant biomass (Prather et al., 2018), nor did Na affect plant diversity or the relative abundance of different functional groups of plants (Prather et al., unpublished data). However, plant quality (i.e., chemistry) did change in response to Na – the relative abundance of N:Na declined with additional Na, and further declined when NP and Na were added in combination (Prather et al., 2018). This increase of Na in plants could have alleviated the grasshoppers from Na limitation, a phenomenon that has been repeatedly shown for herbivores and omnivores (Clay et al., 2017; Kaspari et al., 2017; Welti et al., 2019, 2020; Kaspari, 2020). These changes to plant chemistry could have also altered the diet of the katydids, and the katydids could have changed the relative amounts of plants and prey in their diet. In turn, this could have caused changes to the microbiome communities inside the katydids’ guts. Because the bacterial and fungal communities exhibited distinctly different responses, we discuss below specifics about how these communities changed, and proposed potential investigations to further establish the mechanisms underlying the changes.

### Bacterial Communities

For the gut bacterial communities, there are distinct differences between the effects of added NP and added Na. For NP, it has been observed that soil bacterial and fungal communities similarly respond directly to added NP with increased growth and shifts in community composition (Lv et al., 2017; Nottingham et al., 2018). More specifically, added NP was shown to contribute both directly and indirectly to a decrease in microbial richness and shifts in microbial communities in fertilized soils (Campbell et al., 2010; Zeng et al., 2016). The resulting shifts in community functional traits could affect nutrient cycling by the microbial communities in added NP soil (Leff et al., 2015). Therefore, for our observation where gut bacterial community samples from plots with added NP (NP and NP × Na) were less diverse than ambient controls, one possible interpretation would be that the gut bacterial communities respond to NP addition in a way similar to how the soil bacterial communities respond to NP addition. Furthermore, considering that nitrogen addition to the soil is known to decrease the diversity of plants, as well as animals in the affected area (Heidorn and Joern, 1987; Southon et al., 2013), the connectivity of the NP status in soil, plant, animal, and the gut microbiome of the animals might be stronger than previously recognized and will require additional investigations to establish the relationship.

One specific piece of evidence provided by our study involves Sphingomonadaceae – the only indicator group for added NP. Sphingomonadaceae belongs in the Alphaproteobacteria class and can be found in a variety of habitats, including soil and plant phyllosphere and rhizosphere. On leaf tissues, the abundance of Sphingomonadaceae could be significantly increased by herbivory (Humphrey and Whiteman, 2020). Therefore, it is possible that if NP treatments uniquely stimulated higher herbivory, the higher levels of Sphingomonadaceae would be transferred into the gut of the herbivores. In addition, a more general piece of evidence we observed was the shifts in the bacterial community from relatively high abundance of Proteobacteria and Firmicutes to a predominance of Tenericutes in the NP cohort. Tenericutes are Gram-positive bacteria with members known to have association with plants and animals (Gupta et al., 2018). Therefore, these NP-elicited changes, including the abundance of Sphingomonadaceae or Tenericutes, may be the result of shifts in plant and soil microbiomes in combination with potential changes in insect feeding behaviors.

In contrast to NP treatments, bacterial community diversity increased in hosts collected from added Na plots. Also, the level of diversity is much higher in the NP × Na treatment samples compared to that in the NP treatment samples, suggesting that the effect of added Na is overriding the effect of added NP. If the Na treatment resulted in an increase influx of Na in the host diet, it is possible that the higher diversity is a direct consequence of added Na, perhaps as a stressor to select and enrich for a halophilic or halotolerant community. Alternatively, the higher diversity may be an indirect effect of changing insect behaviors, either sodium seeking or avoidance, that bring *O. vulgare* into contact with a higher variety of microbes as it feeds and scavenges. Salinity has been associated with changes in fitness and abundance of soybean aphids and oviposition choices in tiger beetles (Hoback et al., 2000; Eichele-Nelson et al., 2018). Moreover, in soil communities, increased salinity can lead to increases in richness and diversity of bacterial and fungal community composition (Mohamed and Martiny, 2011; Thiem et al., 2018), observations supporting a direct connection between soil and the animal gut microbiome.

Curiously, a deeper look into the Na indicator group seems to support the environmental Na playing a role in influencing the katydid gut microbiome through influencing soil as well as plant microbial communities. There are several unique taxa observed with added Na gut bacterial communities (both Na added and NP × Na added treatments). Several indicator groups for added Na communities were identified, including *Corynebacterium* – a Gram-positive bacterium in the Actinobacteria phylum. While multiple *Corynebacterium* species are notable human pathogens (Bernard, 2012), other research has identified isolates of this genus that are highly associated with sugarcane rhizosphere with increased soil salinity (Pirhadi, 2018). Phyllobacteriaceae and Streptococcaceae were also indicator groups for added Na and are bacterial families previously identified to be associated with high saline soil microbiomes (de León-Lorenzana et al., 2017; Genderjahn et al., 2018). Listeraceae and Propionibacteriaceae, additional indicator groups for added Na, are also families with species that are capable of growth in relatively high concentrations of Na (Labadie et al., 2003; Liu et al., 2005). Again, despite the limited number of samples in our study, the identification of these indicator groups presents a novel line of inquiry to identify the role of the gut microbiome in host fitness and behavior upon Na perturbations.

Our previous work has indicated that *O. vulgare*, when compared to other related grasshoppers, contains characteristic bacterial communities that may depend on diet or evolutionary lineage (Muratore et al., 2020). While almost nothing is known about the mode of transmission for bacterial communities in *O. vulgare*, it can be assumed that some of these resident bacteria come from diet, environmental contact, and feeding behaviors (Billiet et al., 2016; Krams et al., 2017). A large study investigating over 200 different insect species has indicated that gut bacterial communities among omnivorous insects are more diverse in general than those in strict herbivores or carnivores (Yun et al., 2014). This general conclusion implies that for the omnivorous *O. vulgare*, a shift in host feeding behaviors, perhaps from fewer plants to scavenging or vice versa, may correlate with a shift in the host microbiome. Beyond diet and feeding behaviors, it is also possible that many of the microbes in the *O. vulgare* gut community originate from soil and therefore shifts in gut microbial communities represent shifts in the soil microbial communities. In feeding trials, caterpillars who fed on intact dandelion plants had a microbiome that more closely resembled the soil microbiome than the phytobiome of the dandelion (Hannula et al., 2019). With previous evidence suggesting that both soil and plant microbiomes are affected by changes in environmental nutrients like NP and Na (Lv et al., 2017; Thiem et al., 2018), better understanding of the nutrient levels inside the grasshopper gut (Holmes et al., 2017; Bier et al., 2018) will provide insight into whether changes to gut microbiome are the direct result of changing environmental nutrient status.

### Fungal Communities

The decrease in fungal diversity in both added NP and added Na treatment groups suggests that the gut fungal microbiome in *O. vulgare* is also susceptible to environmental NP and Na perturbations. However, whether this is a direct or indirect relationship is, again, difficult to determine. Increased soil salinity has been indicated as a cause of decreased fungal diversity, especially in estuary soils (Mohamed and Martiny, 2011; Thiem et al., 2018). Therefore, changes in the soil fungal community in response to Na addition may indirectly contribute to the community shifts observed inside *O. vulgare.*

To delve deeper into our data, the number of fungal OTUs shared by all groups (18) was lower than the total number of unique fungal OTUs (51), suggesting a small “core” fungal community in these animals. Some of these shared groups, such as the order Capnodiales, have established associations with insects like scale and aphids (Stephenson, 2012). Other shared taxa, such as genera *Cryptococcus* and *Hannaella*, have been previously associated with plants (Wen et al., 2017). The one unique group distinctive as an indicator of added Na gut fungal communities were members of the hyphomycete genus *Cladosporium*, which is categorized as an osmotolerant mold (Araújo et al., 2020) and are found in a wide range of environmental habitats, including hypersaline waters (Bensch et al., 2012).

The *O. vulgare* gut samples in this study yielded a total of 99 species-level OTUs. However, because taxonomic resolution is still relatively low for fungal communities, many fungal OTUs were unspecified or uncategorized. This in part due to the lack of well-established databases of fungal ITS sequences, and in part due to the convolutions of fungal taxonomy (Tang et al., 2015; Gdanetz et al., 2017). Moreover, historically speaking, research into the microbiome of insects has been biased towards bacterial community characterization, rather than characterization of the whole gut microbiome. This one-domain approach to learning about insect microbiomes has inevitably led to gaps in our understanding of the contributions by other Eukaryotic members in the insect microbiome (Gurung et al., 2019). Also, the characterization of the host-fungal microbiome relationship is largely informed by the studies of insect fungal pathogens or plant fungal pathogens transmitted by insects. Characterization of the fungal microbiome of insects like *O. vulgare* helps us to begin to gain a more comprehensive awareness of these fungal communities and their potential functions.

## Conclusion

Here we demonstrated that abiotic factors, in this case, environmental shifts in soil nutrients like NP and Na, result in changes the gut microbiome of *O. vulgare*. Although our sample number is limited, significant differences in microbial community composition between treatment and control groups were observed, supporting a continuity of microbial communities across soil, plant, animal and raising the hypothesis that nutrient limitation may also exist in the gut microbiome. We further demonstrated that the fungal microbiome of *O. vulgare* responded to environmental perturbations very differently than the co-existing bacterial microbiomes. We identified salt-tolerant genera *Corynebacterium and Cladosporium*, as well as families like Streptococcaceae, Propionibacteriaceae, Phyllobacteriaceae, and Listeriaceae as indicators of insect microbiomes exposed directly or indirectly to increases in environmental sodium. While these results may be considered preliminary in nature, they provide an important insight into insect gut microbiome structure and function upon environmental perturbations. Future studies to better establishing mechanisms contributing to characteristic insect gut microbiomes under the influence of changing ecosystem conditions, such as changing nutrient levels, will help us identify the roles of microbes in sensing and responding to disruptions that may threaten insects or their ecosystems.

## Data Availability Statement

The datasets presented in this study can be found in online repositories. The names of the repository/repositories and accession number(s) can be found below: Dryad Repository, https://doi.org/10.5061/dryad.t4b8gthzp.

## Author Contributions

All authors participated in the writing and preparation of the manuscript. Insect collection and prairie fertilization were performed under the direction of CP. Sample preparation and data analysis were performed by MM.

## Conflict of Interest

The authors declare that the research was conducted in the absence of any commercial or financial relationships that could be construed as a potential conflict of interest.

## Supplementary Material

Supplementary Figure 1 Rarefaction curves for bacteria **(A)** and fungal **(B)** taxa in each sample. Analysis performed in Qiime (v.1.9.1).

Supplementary Figure 2 Species accumulation curves for bacteria **(A)** and fungal **(B)** communities calculated using specaccum function in vegan package of R (v.3.6.2).

Supplementary Table 1 Number of reads per *Orchelimum vulgare* sample..
