# Supplementary figures and images for: Environmental Nutrients Alter Bacterial and Fungal Gut Microbiomes in the Common Meadow Katydid, Orchelimum vulgare
Source: Front Microbiol. 2020 Oct 23;11:557980. doi: 10.3389/fmicb.2020.557980 (PMC7645228; doi:10.3389/fmicb.2020.557980)

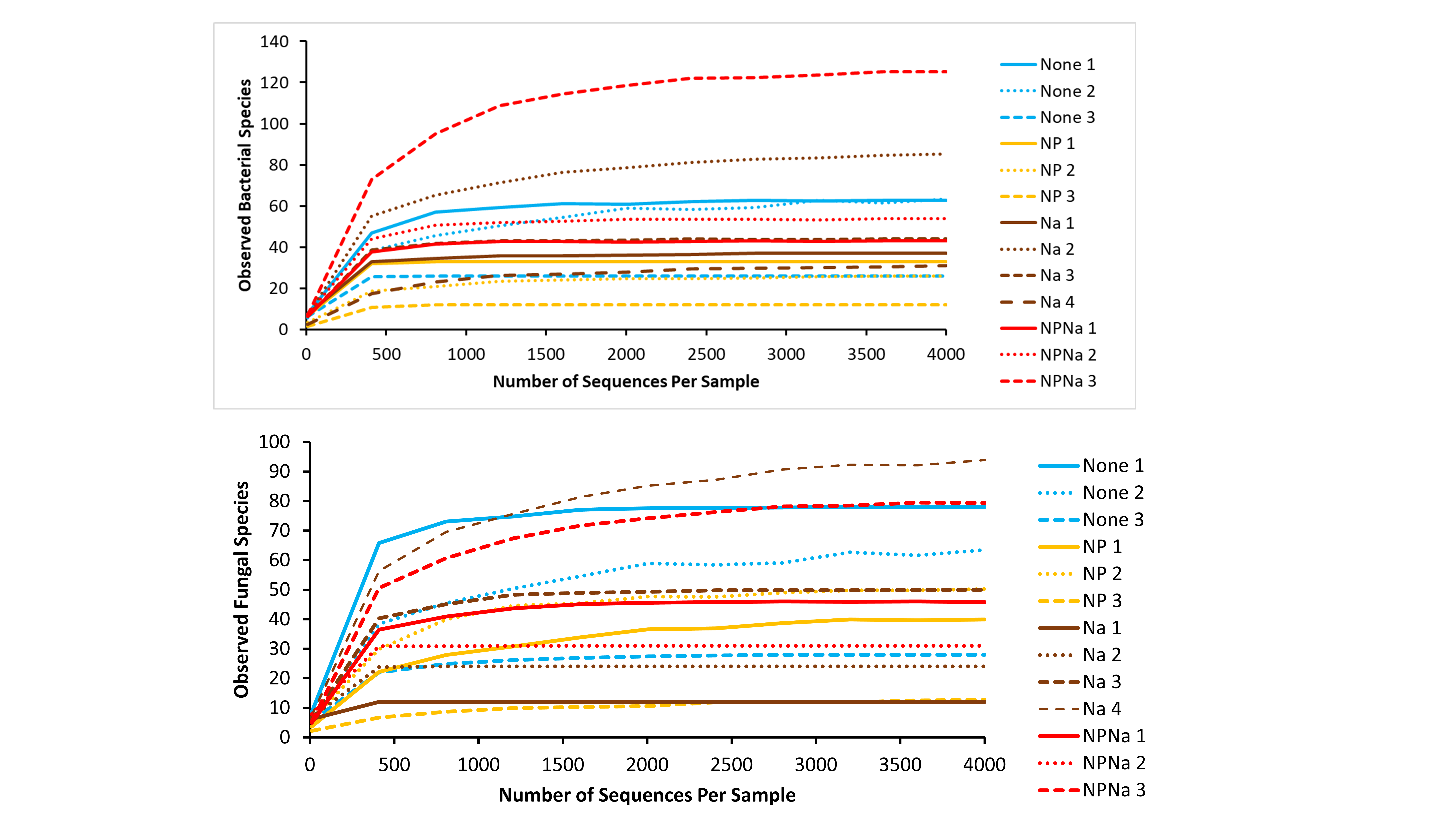

Supplement: Supplementary Figure 1 — Rarefaction curves for bacteria (A) and fungal (B) taxa in each sample. Analysis performed in Qiime (v.1.9.1). [file Image_1.TIF]

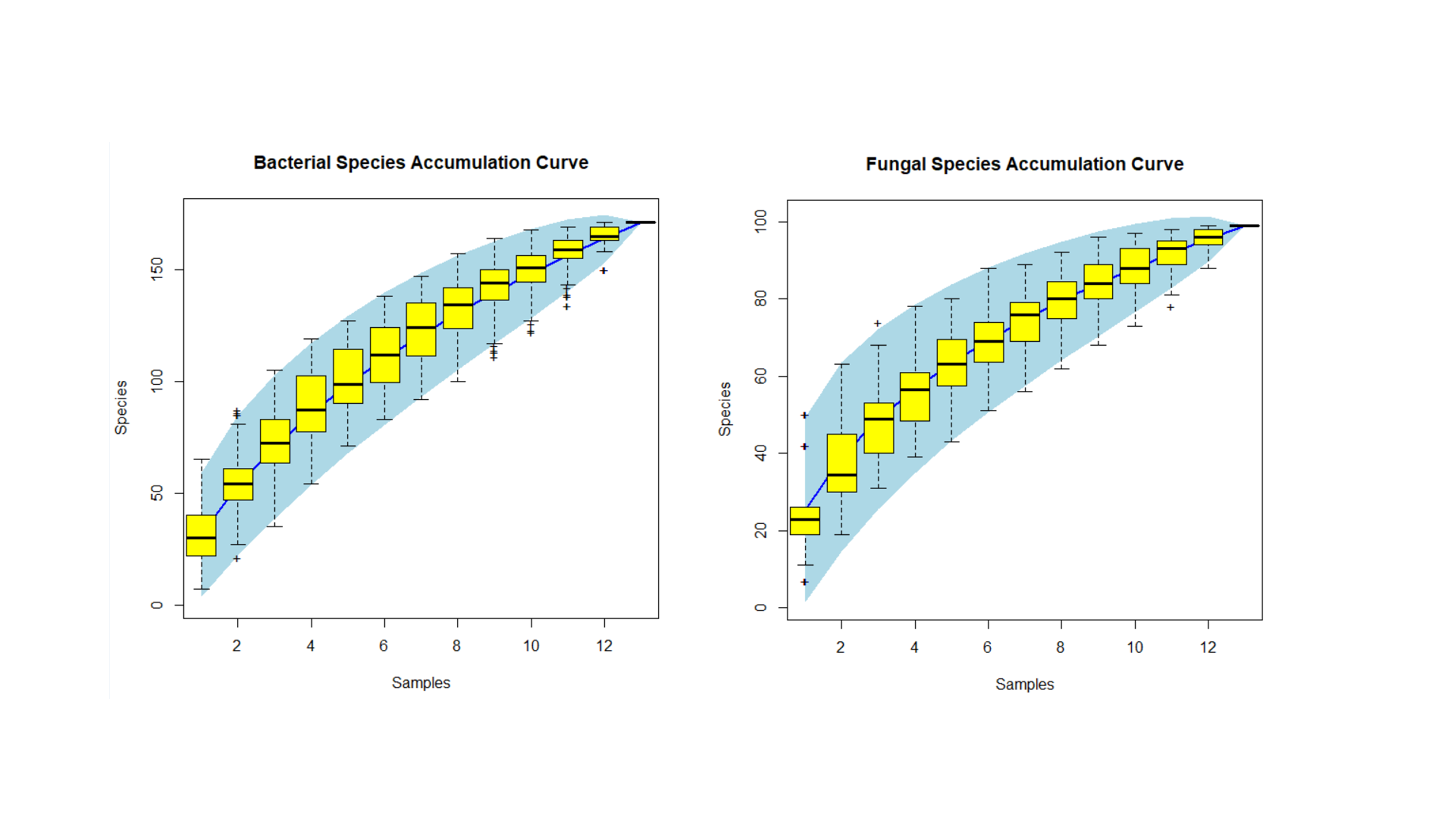

Supplement: Supplementary Figure 2 — Species accumulation curves for bacteria (A) and fungal (B) communities calculated using specaccum function in vegan package of R (v.3.6.2). [file Image_2.TIF]
